# Analysis of food and fluid intake in elite ultra-endurance runners during a 24-h world championship

**DOI:** 10.1186/s12970-020-00364-7

**Published:** 2020-07-11

**Authors:** Chloé Lavoué, Julien Siracusa, Émeric Chalchat, Cyprien Bourrilhon, Keyne Charlot

**Affiliations:** 1 Institut de Recherche Biomédicale des Armées, Unité de Physiologie des Exercices et Activités en Conditions Extrêmes, Département Environnements Opérationnels, 1 place Général Valérie André, 91223 Bretigny-Sur-Orge, France; 2 LBEPS, Univ Evry, IRBA, Université Paris Saclay, 91025 Evry, France

**Keywords:** Ultramarathon, Energy expenditure, Energy balance, Running, Nutrition, Hydration

## Abstract

**Background:**

Properly replacing energy and fluids is a challenge for 24-h ultramarathoners because such unusually high intake may induce adverse effects (gastrointestinal symptoms [GIS] and exercise-associated hyponatremia [EAH]). We analyzed such intake for 12 twelve elite athletes (6 males and 6 females; age: 46 ± 7 years, height: 170 ± 9 cm, weight: 61.1 ± 9.6 kg, total distance run: 193–272 km) during the 2019 24-h World Championships and compared it to the latest nutritional recommendations described by the International Society of Sports Nutrition in 2019. We hypothesized that these elite athletes would easily comply these recommendations without exhibiting detrimental adverse symptoms.

**Methods:**

Ad libitum food and fluid intake was recorded in real-time and energy, macronutrient, sodium, and caffeine intake then calculated using a spreadsheet in which the nutritional composition of each item was previously recorded. GIS, markers of dehydration (body mass modifications, plasma and urine osmolality, and plasma volume; samples obtained 26 h before and just after the race) and EAH (plasma and urine sodium concentrations) were also assessed.

**Results:**

Fluid, energy, and carbohydrate intake of the 11 finishers was 16.4 ± 6.9 L, 35.1 ± 15.7 MJ, and 1.49 ± 0.71 kg, respectively. Individual analyses showed that all but one (for fluid intake) or two (for energy and carbohydrate intake) consumed more than the minimum recommendations. The calculated energy balance remained, however, largely negative (− 29.5 ± 16.1 MJ). Such unusually high intake was not accompanied by detrimental GIS (recorded in 75%, but only transiently [3.0 ± 0.9 h]) or EAH (0%). The athletes were not dehydrated, shown by the absence of significant body mass loss (− 0.92 ± 2.13%) and modifications of plasma osmolality and an increase in plasma volume (+ 19.5 ± 15.8%). Performance (distance ran) positively correlated with energy intake (*ρ =* 0.674, *p =* 0.023) and negatively (*ρ =* − 0.776, *p =* 0.005) with fluid intake.

**Conclusions:**

Overall, almost all of these elite 24-h ultramarathoners surpassed the nutritional recommendations without encountering significant or the usual adverse effects.

## Background

Ultramarathon races (> 42.195 km) have gained popularity in the last two decades [1–3] and are now used as experimental models to assess the effects of sleep deprivation [4, 5] and muscular [6, 7] and cardiac [8, 9] damage. Most of the focus has been on the analysis of nutritional and fluid intake [10, 11]. Indeed, these long efforts induce considerable losses, including those of energy stores, water, and electrolytes. The amount of food and fluid intake is highly suspected to modulate certain causes of fatigue (muscle glycogen content and blood glucose availability) and therefore to influence performance [12]. Moreover, large differences in intake have been observed between finishers and non-finishers [13, 14], suggesting a link between intake and performance, unless gastrointestinal distress reduced energy intake due to a reduced appetite, explaining such withdrawals.

Indeed, replacing such significant losses without overloading an already severely distressed organism (especially the gastrointestinal tract) is a difficult challenge for participants. Indeed, high intake of carbohydrates (particularly hyperosmolar solutions) appears to be the primary nutritional cause of gastrointestinal symptoms (GIS) [15], which occur quite frequently during these races (> 65% of participants) [15] and identified as the main cause of dropping out [16, 17] and reducing overall intake [18, 19]. Moreover, water replenishment using hypotonic fluids may dilute plasma sodium concentrations and cause exercise-associated hyponatremia (EAH) [20, 21]. This is an adverse effect that is quite rare for races below 100 km (prevalence < 3%) [21] but affects approximately 20% (range: 6–51%) of participants in races above 100 km [21, 22], potentially causing a myriad of mild-to-severe symptoms, including GIS (nausea/vomiting) [21]. Regularly updated benchmark recommendations [10, 12, 20] have been proposed to help athletes plan adequate energy, carbohydrate, protein, liquid, and sodium intake to limit the depletion of energy stores and dehydration and thus the occurrence of EAH and GIS. The International Society of Sports Nutrition recently recommended that ultra-endurance athletes should aim for between 450 and 750 mL.h^− 1^ of fluid, 30 and 50 g.h^− 1^of carbohydrate, and 0.67 and 1.67 MJ.h^− 1^ (or 150–400 kcal.h^− 1^) of energy intake [10]. These recommendations are based on a limited number of studies and any assessment of intake during an ultra-endurance event would help in adjusting them.

Observational studies have highlighted 1) high variability in intake among participants [23–25] and 2) a failure to respect recommendations; i.e. actual intake lower than that advised [23, 26, 27]. The reasons for these discrepancies are numerous. Some evidence suggests that athletes are not necessarily aware of such advice or choose to follow their sensations during the race rather than science-based evidence [28]. Another hypothesis is the impossibility to respect these guidelines during the race because of the aforementioned undesirable events [19]. Another hypothesis is that they reach an alimentary limit [29] that impedes increasing intake above a threshold fixed at 2.5 times the resting metabolic rate.

Among the myriad of ultra-endurance races, the 24-h ultramarathon is distinguishable from others as athletes have to repeat a small loop multiple times (6 km for the Glenmore24 [19, 23]) instead of following a one-way route [13, 24, 26, 27]. From a logistical point of view, this implies that food and drink intake is facilitated, since athletes very frequently pass in front of the food supply tents. We recently observed that facilitating food intake during a multi-day expedition in Greenland resulted in adequate energy intake [30]. We thus hypothesized that nutritional and fluid intake would be greater during a 24-h ultramarathon than that during other ultramarathons. To date, only Costa et al. [23] have assessed energy balance during a 24-h ultramarathon in non-elite athletes. They observed that energy intake was less than half of energy expenditure and that most participants failed to adhere to the recommendations, with the exception of carbohydrate intake. In October 2019, the World Championships took place in Albi, France, affording the possibility to assess intake in elite athletes. It has been suggested that intake may increase with the level of performance [23] and thus recommendations are likely to be better followed in this population.

Our main objective was to quantify food and fluid intake (origin of food and fluids and energy, macronutrient, sodium, and caffeine intake) during the 24-h run World Championships in 12 members of the French national team. The second objective was to identify whether these participants respected the latest benchmark recommendations [10] and experienced GIS, signs of dehydration (body mass loss and decrease in urine and plasma osmolality), and EAH. This competition was run in relative comfort, with easy and very frequent access to food and fluid (loops of 1.5 km) on a flat circuit in non-extreme environmental conditions (10–25 °C without rain or wind). Thus, environmental biases and potential stressors were lower than during other ultramarathons.

## Methods

### Nature of the event

**Fig. 1:**
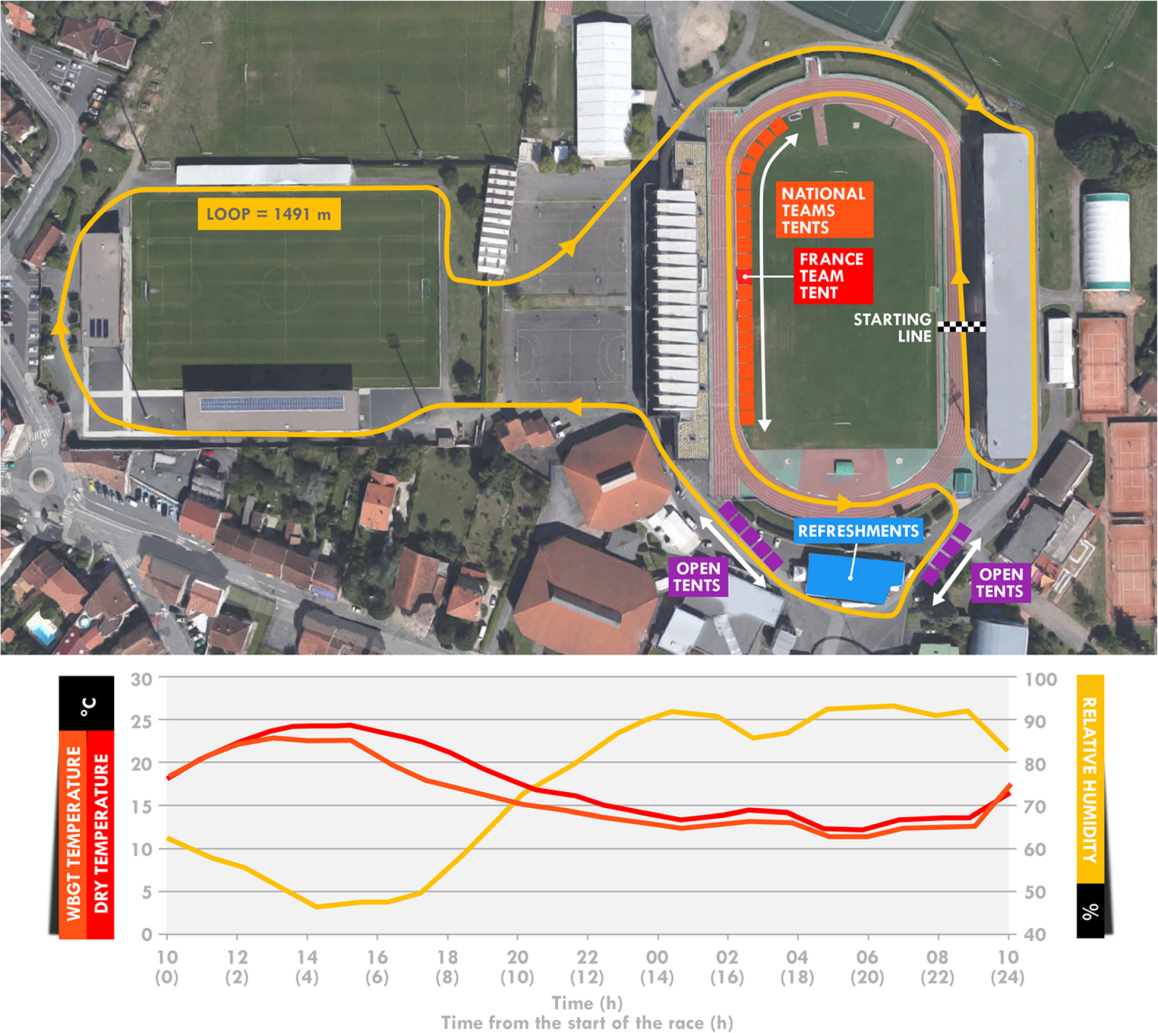
Aerial view of the accommodations of the race loop (**a**) and meteorological conditions (**b**). Open tents were reserved for open athletes (not selected by national teams). The aerial view was extracted from®Google Maps

This study was conducted during the 24-h ultramarathon World Championship held in Albi (France) from October 26–27, 2019. The race consisted of running the greatest distance possible over 24 h (start of the race at 10:00 am the first day). Participants ran on a short loop (1.491 km) combining asphalt (~ 75%) and tartan track (~ 25%) (Fig. 1). The race took place in a mild-to-hot environment, with sunny weather (Fig. 1). The mean dry temperature was 17.4 °C [min-max: 12.2–24.3], wet-bulb temperature 14.2 °C [11.5–17.2], and globe temperature 19.8 °C [11.0–35.1]. The WBGT temperature was 16.0 °C [11.3–23.1]. Relative humidity was 74.0% [47.2–92.7] and the wind speed 0.7 m.s^− 1^ [0.0–2.7]. All weather measurements were made using a weather station (Kestrel Meter 5400 Heat Stress Meter, Birmingham, MI, USA) near the track at a height of 1.2 m and exposed directly to the sun.

### Subjects

Twelve French elite athletes (6 men and 6 women) agreed to participate in this study (see Table 1 for characteristics). The study was conducted in accordance with the Declaration of Helsinki and was approved by the regional ethics committee (CPP Ile-de-France 8, France, registration number: 2019-A02445–52, Etude LemuR). The participants’ written informed consent was obtained after they were informed of the purpose and procedures of the study.

**Table 1:** Participant characteristics

Participant	Performance	Speed	Gender	Age	Height	Weight	Fat mass
#	km	km.h^− 1^	M or F	years	cm	kg	% of body mass
1	272	11.3	M	39	181	75.0	4.2
2	259	10.8	M	53	170	60.0	6.2
3	248	10.3	M	53	172	63.1	8.9
4	241	10.0	F	37	160	42.9	10.1
5	236	9.9	M	46	188	73.8	14.5
6	236	9.8	M	50	175	69.5	9.7
7	222	9.2	F	52	166	53.1	23.5
8	219	9.1	F	45	160	51.9	19.1
9	209	8.7	F	46	160	62.9	18.6
10	201	8.4	F	31	171	58.0	12.0
11	193	8.0	F	52	169	61.4	22.2
12^a^	133	5.5	M	54	173	68.2	10.0
Mean	231	9.6		46	170	61.1	13.5
SD	24	1.0		7	9	9.6	6.5

^a^This participants abandoned and was therefore excluded from the nutritional analyses. The means were therefore calculated without him

### Design

Participants were free to bring their personal food and drink. Energy and the macro- and micronutrient composition of all items were registered in the days before the event. Four participants using self-manufactured foods (less than 10 items in total) were asked to provide their recipes to establish the food composition.

Food and/or drinks were handed out as the participants passed in front of the France team tent (in red in Fig. 1) according to an individual nutritional program provided to the team crew prior to the race. Even if no intake was programmed, food and drinks from their selection were still available on a tray to allow the participant to pick one of them if necessary. Participants were then free to modify their program and ask for unplanned or common foods. Indeed, a selection of food and drinks was available in large amounts for all participants. All consumed food and drinks were registered, along with the amount consumed (in g or ml). To do so, team members used an individual chart displaying programmed items and quantities consumed during the run. When an item was consumed according to the program (i.e. consumed at the intended loop), it was circled. If the quantity differed, it was corrected using a blank column. Finally, when unplanned items were consumed, it was recorded, along with its quantity, in the same blank columns. The same two members of the team were assigned to four participants during the entire race.

The refreshments tent (in blue in the Fig. 1) provided a complementary source of food and drinks, providing mostly water, cake, fruit, and mashed potatoes. Participants were asked to indicate the amounts consumed after the race.

Urine and blood samples were obtained one-day before and immediately after the race for biological analysis (urine and plasma osmolality and sodium concentrations).

### Methodology

#### Food intake

The total food and fluid intake were calculated using spreadsheets (Excel for Office 365, Microsoft, Redmond, WA, USA), a composition table of each food and drink consumed, and the timing of their intake. Foods were separated into soft (all food that did not require chewing) and solid items. Fluids were separated into water and caloric fluids (with energy content). Energy and macronutrient intake were also determined. Relative intake was then calculated. Thus, total energy intake was first split into the nature of the foods (soft and solid foods and caloric drinks) and then into macronutrient intake (carbohydrate, fat, and protein). Finally, sodium and caffeine intake were also calculated.

Energy, carbohydrate, protein, and fluid intake were compared to the latest benchmark recommendations [10]: 150–400 kcal.h^− 1^ (0.67–1.67 MJ.h^− 1^), 30–50 g.h^− 1^, 5–10 g.h^− 1^, and 450–750 mL.h^− 1^, respectively.

All food and drinks given to the participants were compatibilized in the calculation. When a bottle was returned unfinished, the unconsumed volume was withdrawn. We did not witness whether the participants consumed all that was handed out. They indicated to us that they ate all that was picked up at the tent. However, it is possible that a small amount was not consumed for multiple reasons (part of the food thrown away with the wrapper, water used to spray themselves, etc.) and this overestimation was hard to assess. Another source of inaccuracy was the accounting of items selected from the refreshments tent. Even if the recollection occurred just after the race, it is possible that the reported amounts diverged slightly from reality. Nevertheless, the amounts of items originating from the refreshments tent were marginal and the degree of imprecision theoretically insignificant.

#### Energy expenditure

Given the extreme competitive context of the race, participants refused to wear accelerometers, as in the study of Costa et al. [23], or heart rate monitors. An alternative solution was the use of an algorithm based on weight, resting heart rate, and running speed [31] to estimate energy expenditure. The running speed for each participant was retrieved from the organization (https://www.breizhchrono.com/detail-de-la-course/crs_id/13092/ for men and https://www.breizhchrono.com/detail-de-la-course/crs_id/13094/ for women). We acknowledge a certain margin of error, since, in addition to the internal degree of inaccuracy of the algorithm, the impact of accumulated fatigue and weather was not considered by the model. However, it has been shown that running speed is as accurate as heart rate for the assessment of energy expenditure [31].

#### Symptomology

Each hour, participants were asked by the team physician whether they experienced GIS or other symptoms (e.g. articular or muscular pain). They were informed before the race of the list of GIS (difficulty swallowing, belching, acid reflux, heartburn, nausea, vomiting, abdominal pain, bloating, flatulence, urge to defecate, diarrhea, and constipation) to facilitate the identification of these symptoms during the race. Their occurrence was therefore noted in real-time. A few hours after the race, these observations were cross-checked with the athletes.

#### Biological measurements

Blood was drawn from the antecubital vein 26 h before the race (between 7:00 and 9:00 am), and within 30-min of finishing. Blood was collected into two separated tubes (Becton Dickinson, Franklin Lakes, USA), one EDTA (5 mL) and one Lithium Heparin (5 mL). Tubes were conserved at 4 °C and plasma was separated within 1 h by centrifugation (2000 x g, 10 min). Participants also provided urine samples at each time point in sterile polypropylene tubes (30 mL).

The presence of urine ketone bodies was detected immediately after collection using a urinary dipstick (Multistix 10 SG Urinalysis, Reagent Strips, Siemens Healthineers, Erlangen, Germany) and a Clinitek Status+ analyzer (Siemens Healthineers, Erlangen, Germany). Four concentrations could be obtained: 0, 5, 15, or 40 mg.dL^− 1^. Plasma and urinary sodium, potassium, urea, and glycaemia were measured using a Roche Cobas c501 (Roche Diagnostics, Meylan, France). Plasma and urinary osmolality were calculated as follows [32, 33]:

$$ \mathrm{Plasma}\ \mathrm{osmolality}\ \left(\mathrm{mOsmol}.{\mathrm{kg}}^{-1}\right)=1.9\ \mathrm{x}\ \Big(\left[{\mathrm{Na}}^{+}\right]+\left[{\mathrm{K}}^{+}\right]+\left[\mathrm{glucose}\right]+0.5\ \mathrm{x}\ \left[\mathrm{urea}\right]+5 $$

$$ \mathrm{Urinary}\ \mathrm{osmolality}\ \left(\mathrm{mOsmol}.{\mathrm{kg}}^{-1}\right)=\left(2\ \mathrm{x}\ \left(\left[{\mathrm{Na}}^{+}\right]+\left[{\mathrm{K}}^{+}\right]\right)+0.9\ \mathrm{x}\ \left[\mathrm{glucose}\right]+0.5\ \mathrm{x}\ \left[\mathrm{urea}\right]\right)\ \mathrm{x}\ 0.985 $$

The hematocrit and hemoglobin concentrations were measured (XN-2000, Sysmex, Villepinte, France) and used to estimate alterations of plasma volume [34]. This technique has already been used in a previous 24-h ultramarathon [23].

### Statistical analysis

All data are presented as the means ± standard deviation throughout the manuscript. In the text, the range (minimum value – maximum values) is sometimes presented inside brackets and individual data are also displayed in the Figures. Statistical analyses were performed to assess the biological changes between before and after the race. As the data were not normally distributed, according to Shapiro-Wilk tests, we performed Wilcoxon tests for paired data. The level of association between temperature and intake was assessed using Spearman’s rank correlation coefficient (*ρ*). Significance was defined as *p <* 0.05. Analyses were performed using STATISTICA software (v10, Statsoft, Tulsa, OK, USA).

## Results

### Race details and symptomology

Among the 12 participants, one abandoned during the 13th hour (participant #12) and was therefore withdrawn from the nutritional and biological analyses.

The mean estimated energy expenditure was 64.6 ± 12.1 MJ [50.9–88.4 MJ] and the body mass modification 568 ± 1249 g [− 2500 − + 1249 g; not significant].

Eight participants (67%) experienced at least one GIS (nausea = 4, difficulty to swallow = 3, diarrhea = 2, and vomiting = 1; detailed in Table 2). Muscular pain was observed for five participants. These were transient and localized to the lower limbs in participants #3 and 5. Participants #9 and 11 suffered from lower back pain (lumbar region) and had to drastically reduce their pace until the end of the race. Participant #12 mostly attributed his abandon to thigh pains. Participant #6 fainted during the last hour of the race and was not able to finish.

**Table 2:** Symptoms declared by the participants during the race

	Gastrointestinal symptoms (GIS)					Other symptoms	
Participant	Difficulty to swallow	Nausea	Vomiting	Diarrhea	All GIS	Muscular pain	Fainting
#1	0	0	0	0	0	0	0
#2	0	0	0	0	0	0	0
#3	1 (3)	0	0	0	1 (3)	3 (3–3-6)	0
#4	0	1 (2)	0	0	1 (2)	0	0
#5	1 (2)	1 (2)	0	0	2 (2–2)	1 (2)	0
#6	0	0	0	0	0	0	1 (0.5)^a^
#7	0	0	0	1 (3)	1 (3)	0	0
#8	1 (3)	1 (4)	0	0	2 (3–4)	0	0
#9	0	0	0	0	0	1 (12)	0
#10	0	1 (3)	0	0	1 (3)	0	0
#11	0	0	0	1 (5)	1 (5)	1 (5)	0
#12	0	0	1 (3)	0	1 (3)	1 (6)	0
Total^b^	3	4	1	2	8	5	1
%	25	33	8	17	67	42	8

The number of occurrence for each participant is indicated followed by the duration (in h) of each occurrence in brackets

^a^Fainting occured during the last hour of the race and lasted approximately 30 min until the end of the race

^b^Total number of participants experiencing the same symptom. The number of individual occurrences was not considered for the calculation

### Mean intake analyses

**Fig. 2:**
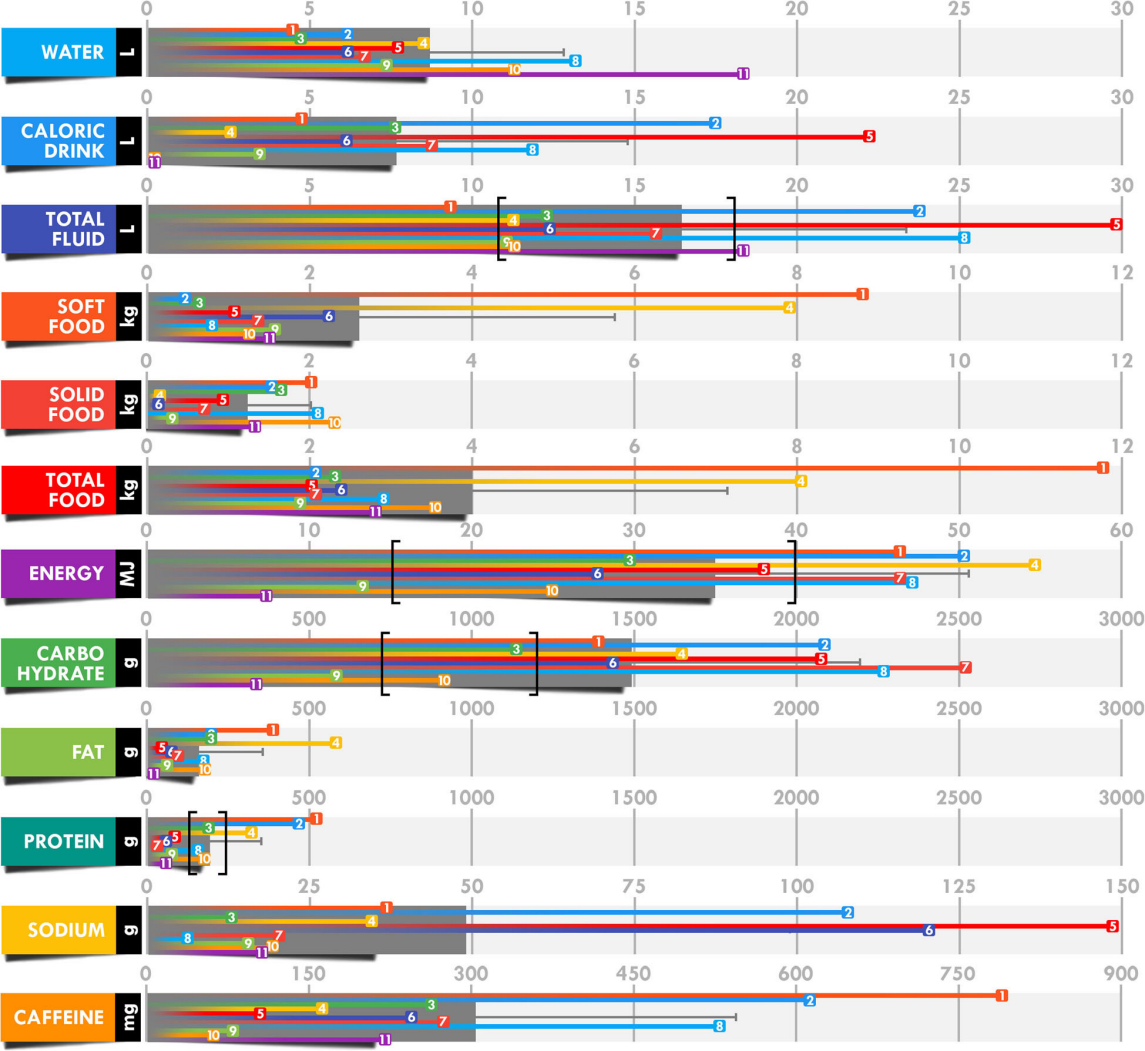
Mean and individual total intake during the 24-h race. Each numbered colored square refers to a single participant. The grey bar indicates the mean and black brackets define the recommendations [10].

**Fig. 3:**
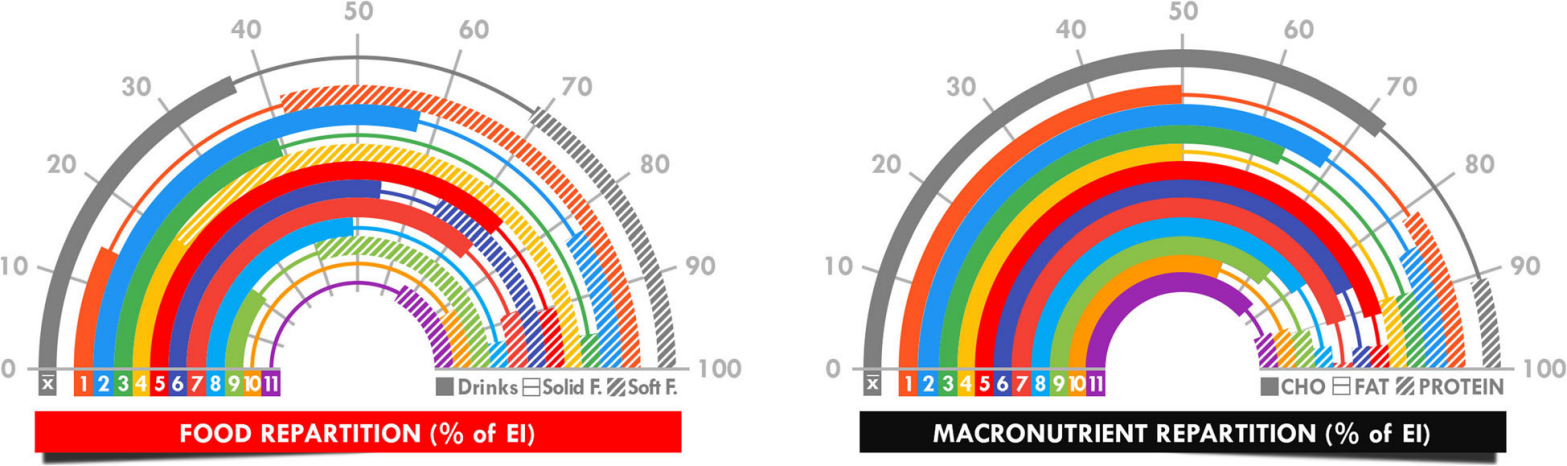
Dissection of total energy intake using food (**a**) and macronutrient (**b**) repartition. Each individual repartition is indicated by colored bars (filled bar, line, and hatched bar) and the mean repartition (x̄) by the grey bar

All individual and mean total intake values are presented in Fig. 2, mean corrected intake for body mass and mean intake rate in Table 3, and relative contribution of fluids and foods and of carbohydrate, fat, and protein in the total energy intake in Fig. 3.

**Table 3:** Intake and intake rate corrected for body mass

	Mean	SD	Minimum	Maximum
Total fluid (ml.kg BM^−1^)	274	115	123	484
Total fluid (ml.h^−1^)^a^	685	290	385	1250
Total food (g.kg BM^−1^)	65	55	28	188
Total food (g.h^− 1^)	159	132	79	491
Energy (kJ.kg BM^−1^)	606	34	119	1278
Energy (kJ.h^−1^)^a^	1463	654	305	2284
Carbohydrate (g.kg BM^− 1^)	25.5	14.0	5.4	47.6
Carbohydrate (g.h^−1^)^a^	62.2	29.6	13.9	105.4
Fat (g.kg BM^−1^)	3.3	3.7	0.3	13.6
Fat (g.h^−1^)	7.7	7.0	0.9	24.3
Protein (g.kg BM^−1^)	3.2	2.8	0.5	7.8
Protein (g.h^−1^)^a^	8.0	7.1	1.2	21.7
Sodium (mg.kg BM^−1^)^b^	765	719	125	2013
Sodium (mg.h^−1^)^b^	2054	2111	271	6189
Caffeine (mg.kg BM^−1^)^c^	5.04	3.77	1.01	10.9
Caffeine (mg.h^−1^)^c^	12.8	10.1	2.4	32.9

^a^Latest benchmark recommendations for fluid, energy, carbohydrate, and protein intake [10]: 450–750 mL.h^−1^, 670–1670 kJ.h^− 1^, 30–50 g.h^− 1^, and 5–10 g.h^− 1^, respectively

^b^In similar studies, sodium intake was observed between 158 and 246 mg.kg BM^− 1^ and 493 and 671 mg.h^− 1^ [13, 14, 35]

^c^Although there are no specific recommendations, it has been advised to repeat doses of 50 mg·h^− 1^ only during the night “when circadian rhythms are likely to be affected” [10]. BM = body mass

**Fig. 4:**
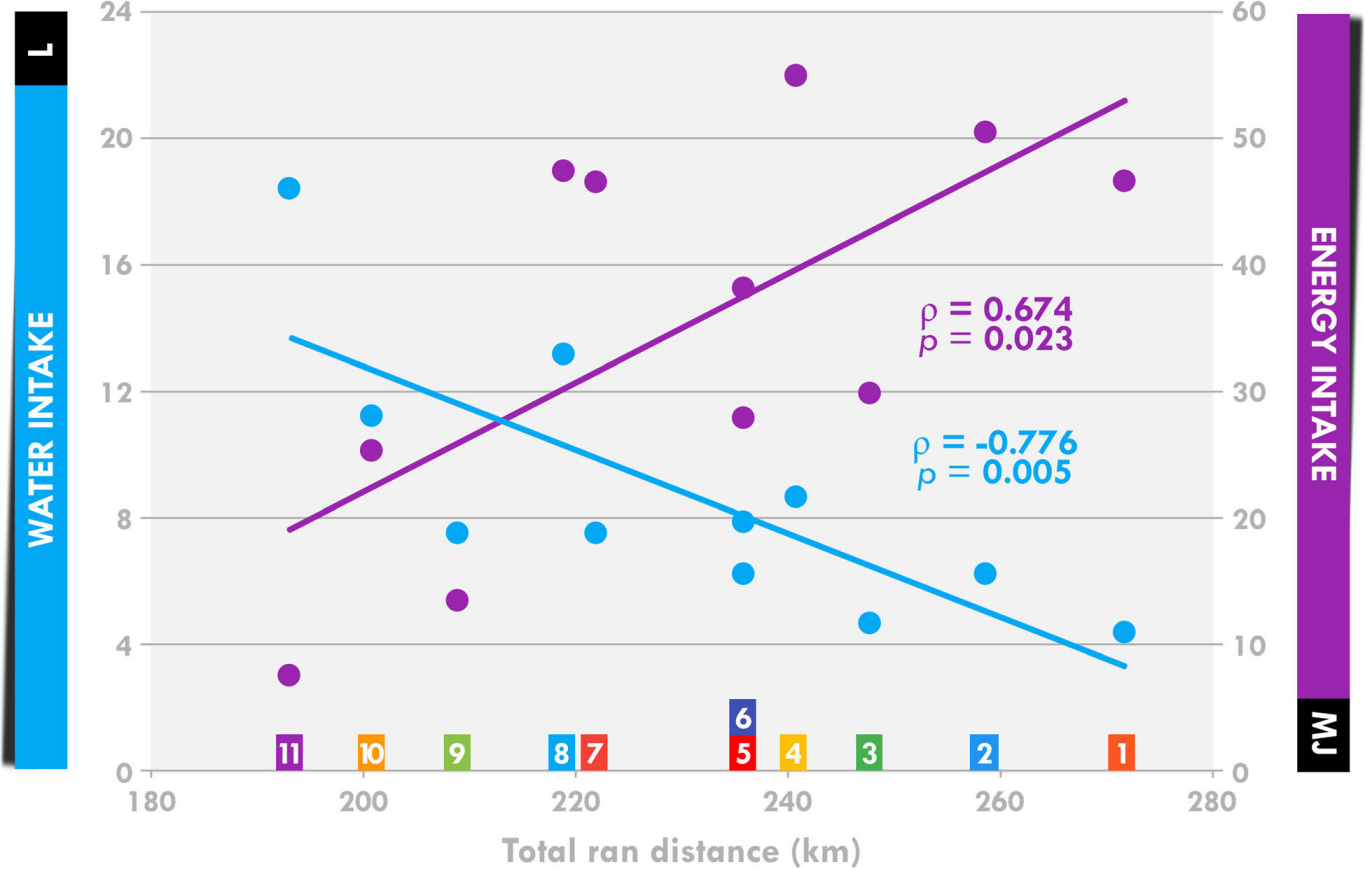
Correlation between performance (total distance ran) and water and energy intake. Each numbered colored square refers to a single participant

There was a negative relationship between performance (total ran distance) and total water intake or total water intake per kg (*ρ =* − 0.756, *p =* 0.007) and a positive relationship between performance and total energy intake (Fig. 4).

### Blood and urine analysis

**Fig. 5:**
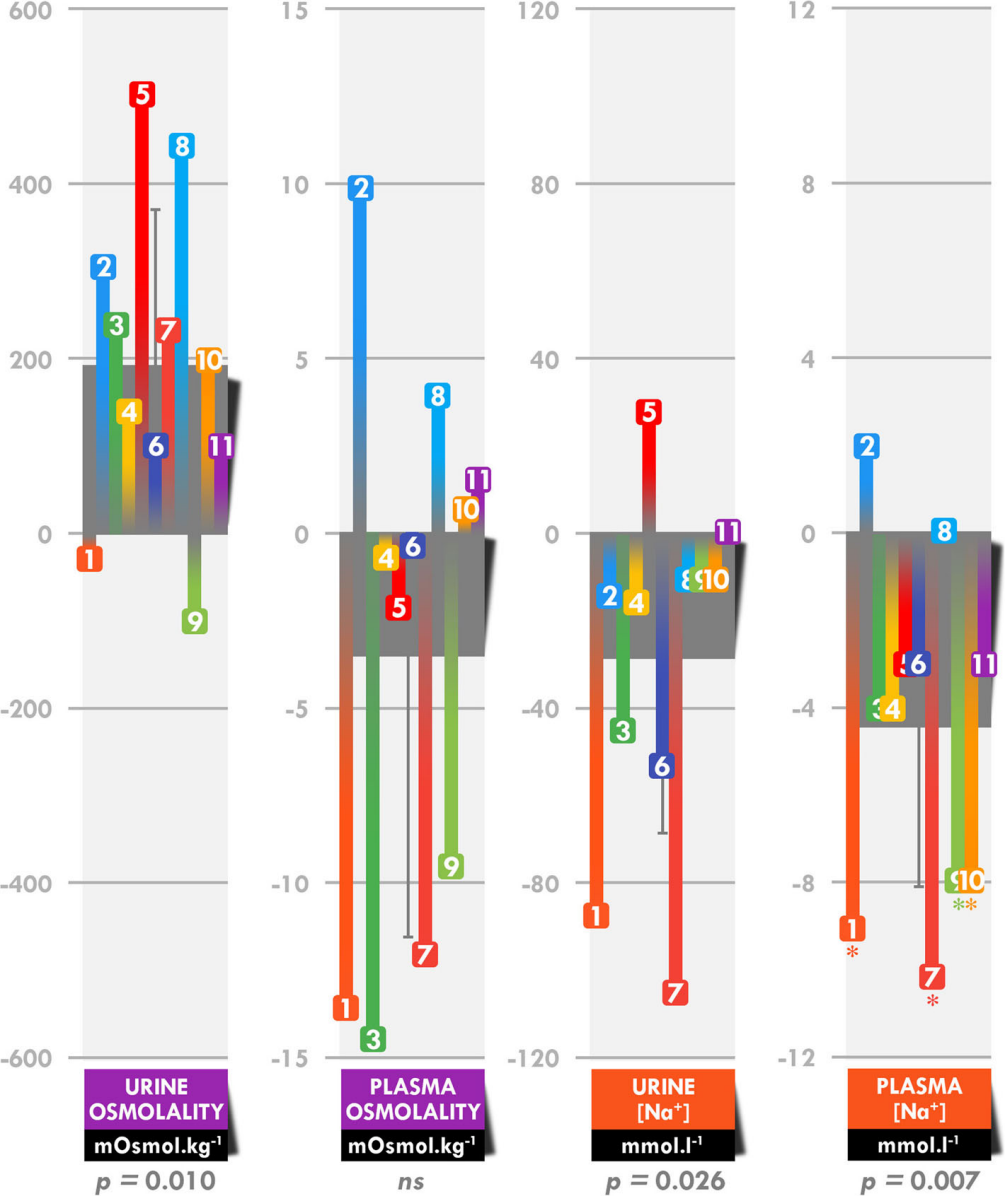
Absolute modifications in urine and plasma osmolality and sodium concentrations. Each numbered colored square refers to a single participant and the grey bar indicates the mean. *These participants reached the threshold for asymptomatic exercise-associated hyponatremia (135 mmol. L^− 1^) [36].

The hematocrit decreased by 13.2 ± 6.7% [− 26.2 – − 5.0%] (*p <* 0.001), whereas the hemoglobin concentration was not significantly modified. Plasma volume increased by 19.5 ± 15.8% [− 0.8 − + 50.8%] (*p =* 0.002). Urine osmolality increased by 40.4 ± 39.4% (561 ± 159 vs 754 ± 186 mOsmol.kg^− 1^; *p =* 0.010) between before and after the race, whereas plasma osmolality was not modified (288.7 ± 3.0 vs 285.3 ± 7.3 mOsmol.kg^− 1^) (Fig. 5). Urinary and plasma sodium concentrations decreased by 27.5 ± 31.1% (85 ± 41 vs 56 ± 30 mmol. L^− 1^; *p =* 0.026) and 3.2 ± 2.7% 141 ± 2 vs 137 ± 3 mmol. L^− 1^; *p =* 0.007), respectively, between before and after the race.

Modifications of osmolality and sodium concentrations correlated with fluid intake. Total fluid intake was positively associated with the post-race plasma sodium concentration (*ρ =* 0.726, *p =* 0.011) and absolute (*ρ =* 0.728, *p =* 0.011) and relative (*ρ =* 0.773, *p =* 0.008) changes in the plasma sodium concentration. Water intake was positively associated with changes in the absolute (*ρ =* 0.694, *p =* 0.018) and relative (*ρ =* 0.779, *p =* 0.005) urine sodium concentration. Finally, total fluid (*ρ =* 0.791, *p =* 0.006) and caloric drink intake (*ρ =* 0.720, *p =* 0.013) was associated with changes in absolute urine osmolality.

Urine ketone body concentrations were null in all participants in pre-race measurements. After the race, they were still not detected in two participants (#5 and 11). Concentrations were estimated to be 5, 15, and 40 mg.dL^− 1^ in two (#4 and 6), four (#3, 8, 9, and 10), and three participants (#1, 2, and 7), respectively.

## Discussion

During the most prestigious 24-h ultramarathon, the levels of energy (up to 11 times the resting metabolic rate), carbohydrate, and fluid intake were globally higher than those of any previously documented ultramarathons, without inducing major detrimental GIS and/or direct noticeable decreases in performance. Logically, almost all participants managed to reach current nutritional and hydration recommendations, despite individualized and different nutritional programs. Indeed, all but one participant drank sufficient fluids (> 450 mL.h^− 1^) and all but two respected the lower energy (> 0.67 MJ.h^− 1^ or 150 kcal.h^− 1^) and carbohydrate (> 30 g.h^− 1^) intake recommendations. Moreover, dehydration and EAH markers were judged to be unaffected in the context of such an unusual and extreme effort, supporting the beneficial effects of following recommendations.

Reaching an energy balance in ultramarathon is illusory, given the amount of energy expended. In their review, Nikolaidis et al. [11] showed that energy deficits are recurrent and can reach up to 2.4 MJ.h^− 1^. This was also true of our study, as we found an energy deficit of approximately 30 MJ (or 1.25 MJ.h^− 1^), corresponding to 45% energy expenditure, despite very high rates of energy and carbohydrate intake (1.46 MJ.h^− 1^ and 62 g.h^− 1^). The presence of ketone bodies in the urine after the race in most participants, also found in a previous study [23], clearly indicates an inadequate rate of the provision of exogenous substrate (especially carbohydrates). Comparisons with swimming, cycling, or triathlon events would be inappropriate due to the large differences in food availability and the facility of intake compared to that with running. Moreover, multiday running events that include periods of sleep minimize the energy deficit, as efforts are not continuous [36] and shorter events are characterized by lower rates of energy intake than longer races [27, 37]. Thus, comparisons are more appropriate with running races lasting approximately 24 h, such as the Javelina Jundred (161 km on a desert trail, mean running duration: 22.5 h) [18], the Western States Endurance Run (161 km; mean running duration: 27.0 h) [13], a 160-km trail race (mean running duration: 24.3 h [38] and 26.2 h [14]), and Glenmore24 Trail Race (24 h; 122–208 km) [23]. The mean rates of energy (1.03, 1.28, 1.05, 1.13, and 0.83 MJ.h^− 1^, respectively) and carbohydrate (47, 66, 54, 54, and 37 g.h^− 1^, respectively) intake were lower than those in the present study. Given the high level of intake, it is not surprising that the energy deficit of this study (30 MJ or 45% of energy expenditure) was lower than that reported in the study of Costa et al. [23], in which they observed a 35 MJ deficit, corresponding to 64% energy expenditure.

There are two principle explanations for the very high levels of observed intake: the configuration of this 24-h World Championship and the level of performance of the studied sample. Concerning the first, the supply of food and fluid was facilitated by the large number of times (between 129 and 182) the participants were able to pass in front of the national team tent, therefore multiplying their potential possibilities for consumption and allowing them to more easily adapt their intake as needed. The second explanation concerns their elite level. The first argument is based on the large difference between the only two studies with a 24-h ultramarathon as an experimental model. We found 76% higher energy intake and 68% higher carbohydrate intake than for the participants of the study of Costa et al. [23]. Although the duration of the events was similar, the possible occasions for consumption were rarer (6-km vs 1.5-km loop) and the terrain was different (mix of off-road terrain, including trails, paths, and grasslands vs asphalt and tartan), these differences potentially explaining a small part of the difference in intake. However, the level of the participants was very different. To substantiate this hypothesis, we observed a link between the performance (distance travelled) and the level of energy intake. Higher rates of energy intake for finishers relative to those of non-finishers [13] and for fast runners compared to slow runners [23] have also been previously reported (the latter difference only almost significant). However, implying that maintaining a high level of intake increases performance is very hasty. The explanations are indeed multiple (better nutritional program, larger amount of energy expended, more passages in front of the tent, etc.), but the most well-documented in the field of ultra-endurance concerns the occurrence and management of GIS.

The occurrence of GIS (75%) in this sample was concordant with the results of similar studies, which reported GIS for 65 to 96% of cases [13, 16, 18, 23, 38], nausea/vomiting being the most frequent symptom [13, 14, 16]. These adverse effects may be very problematic for athletes, as most of the non-finishers of a 161-km ultramarathon (between 23 and 36%) attributed their abandon to nausea/vomiting. The etiology of GIS, while not fully elucidated, is surely multifactorial, with physiological (reduction in splanchnic blood flow) and mechanical factors (pounding and jostling during running) as the main and direct causes [13, 15, 16]. Moreover, the large and unusual amount of food intake during ultramarathons may overload an already severely distressed gastrointestinal tract [10]. The high intake of carbohydrates (particularly hyperosmolar solutions) appears to be the primary nutritional cause of GIS [15]. This hypothesis has been occasionally verified for ultra-endurance running races lasting close to 24 h, with higher carbohydrate intake for participants suffering from GIS [14], but most studies have failed to observe an evident association between the levels of energy or carbohydrate intake and the occurrence of GIS [13, 23, 38]. In the present small elite sample, the episodes of GIS were transient and did not cause major decreases in performance or dropping out, meaning that the athletes tolerated the very high level of energy intake, especially carbohydrates. This may be explained by the training level of these athletes. Indeed, trained athletes are known to tolerate high levels of carbohydrate intake during running [15]. First, certain individuals are predisposed to suffer less from GIS than others [39] and it is possible that elite ultra-endurance athletes come from a pool of less affected individuals. Second, habituation to high-carbohydrate diets during training and competition (i.e. “gut training”) may reduce GI stress through the enhancement of exogenous glucose oxidation [40]. Laboratory studies in which the effects of various levels of energy intake on performance are assessed would improve our understanding of the importance of aiming to maintain high rates of exogenous energy supply. Indeed, such a study was conducted on a single participant [41] and larger psychophysiological disturbances during a laboratory-simulated multistage ultramarathon were observed when energy intake covered only 48% of energy expenditure compared to a well-balanced diet (96%).

Replacement of water and electrolytes lost through sweat evaporation, urination, and respiration is also a difficult challenge for ultramarathoners. With losses estimated to be between 600 and 860 mL.h^− 1^ during 100- to 120-km ultramarathons [24, 25], water replenishment may reach up to 20 L during a 24-h race. However, as a large amount of water is generated by energy oxidation [42], “proper hydration” may be reached in conceding body mass loss exceeding 2% in ultra-endurance races [20]. Maintaining body mass equilibrium may therefore induce hyperhydration, potentially causing a “burdensome gastric load and unabsorbed fluid” in the intestines [20], magnifying the occurrence of GIS and the risk of EAH [20, 21]. Mean fluid intake and body mass loss in the present study (685 mL.h^− 1^ and 0.9%, respectively) were concordant with that previously reported for similar races (790 [18], 747 [13], 684 [38], 740 [14], and 379 [23] mL.h^− 1^ and 0.8–1.9 [18], 3.0 [13], 2.1 [38], 0.6 [14], and 2.0 [23]%, respectively). Although the level of fluid intake was mostly in agreement with recommendations (between 450 and 750 mL.h^− 1^) [10], hyperhydration appeared to be common, given the relatively low body mass loss. It was therefore not surprising to observe a large ~ 20% increase in plasma volume in the present study, double of that reported in the study of Costa et al. [23], in which fluid intake and hyperhydration were less pronounced. Our participants were however spared from EAH. Indeed, although mean plasma sodium concentrations decreased, none of them reached that of symptomatic EAH [35]. Moreover, a positive relationship between fluid intake and post-race plasma sodium concentration suggests that those who ingested the most fluids were the ones who most limited any potential EAH. As this relationship is usually the opposite [14], it is possible that the level of sodium intake through fluids, as well as food, reduced the risk of EAH. Indeed, mean sodium intake was calculated to be 49 g, 2.7 higher than previously observed [18]. Nevertheless, this hypothesis needs to be verified, as sodium supplementation has been shown to inefficiently maintain appropriate hydration [43] or prevent EAH, even in the presence of hyperhydration [20]. In addition, we found a negative relationship between performance and total water intake, suggesting that hyperhydration may not be an efficient strategy, regardless of the absence of EAH. However, it is difficult to know whether such higher water intake in poorer performing athletes is a result of an inadequate hydration program or attempts to calm an excessive sensation of thirst.

## Conclusions

In this study, we observed that elite 24-h ultramarathoners largely respected the latest recommendations by reaching higher levels of energy and carbohydrate intake than in previous 24-h-like ultramarathons. Such high intake was not accompanied by impeding GIS or symptomatic EAH, suggesting a high level of tolerance. Individual-level analysis revealed very different nutritional and hydration strategies and showed that a few participants did not achieve adequate nutrition. Finally, high rates of energy intake and low rates of fluid intake were associated with performance. However, the interpretation and importance of these correlations are yet to be elucidated.

## Data Availability

The datasets used and/or analysed during the current study are available from the corresponding author on reasonable request.

## Abbreviations

EAH: Exercise-associated hyponatremia
GIS: Gastrointestinal symptoms
