# Correction to: Analysis of food and fluid intake in elite ultra-endurance runners during a 24-h world championship

**DOI:** 10.1186/s12970-021-00427-3

**Published:** 2021-04-21

**Authors:** Chloé Lavoué, Julien Siracusa, Émeric Chalchat, Cyprien Bourrilhon, Keyne Charlot

**Affiliations:** 1 Département Environnements Opérationnels, Institut de Recherche Biomédicale des Armées, Unité de Physiologie des Exercices et Activités en Conditions Extrêmes, 1 place Général Valérie André, 91223 Bretigny-Sur-Orge, France; 2 LBEPS, Univ Evry, IRBA, Université Paris Saclay, 91025 Evry, France

**Correction to: J Int Soc Sports Nutr 17, 36 (2020)**

**https://doi.org/10.1186/s12970-020-00364-7**

The original article [1] contained some minor errors in the reporting of sodium intake of three participants during the race.

The final paragraph of the Discussion section in the original article has been updated to correct the initial reporting of sodium intake from 49 g to 20.9 g; associated errors in Fig. 2 and Table 3 have also been corrected.
